# Supplementary material for: Strong vibrational coupling in room temperature plasmonic resonators
Source: Nat Commun. 2019 Apr 4;10:1527. doi: 10.1038/s41467-019-09594-z (PMC6449381; doi:10.1038/s41467-019-09594-z)
Supplement: Supplementary file 1 — Supplementary information [file 41467_2019_9594_MOESM1_ESM.pdf]

Supplementary Information

Strong Vibrational Coupling in Room Temperature Plasmonic Resonators

Junzhong Wang[1], Kuai Yu[1], Yang Yang[1], Gregory V. Hartland[2], John E. Sader[3], Guo Ping Wang[1]

[1]College of Electronic Science and Technology, Shenzhen University, Shenzhen 518060, China

[2]Department of Chemistry and Biochemistry, University of Notre Dame, Notre Dame, IN 46556, USA

[3]ARC Centre of Excellence in Exciton Science, School of Mathematics and Statistics, The University of Melbourne, Victoria 3010, Australia

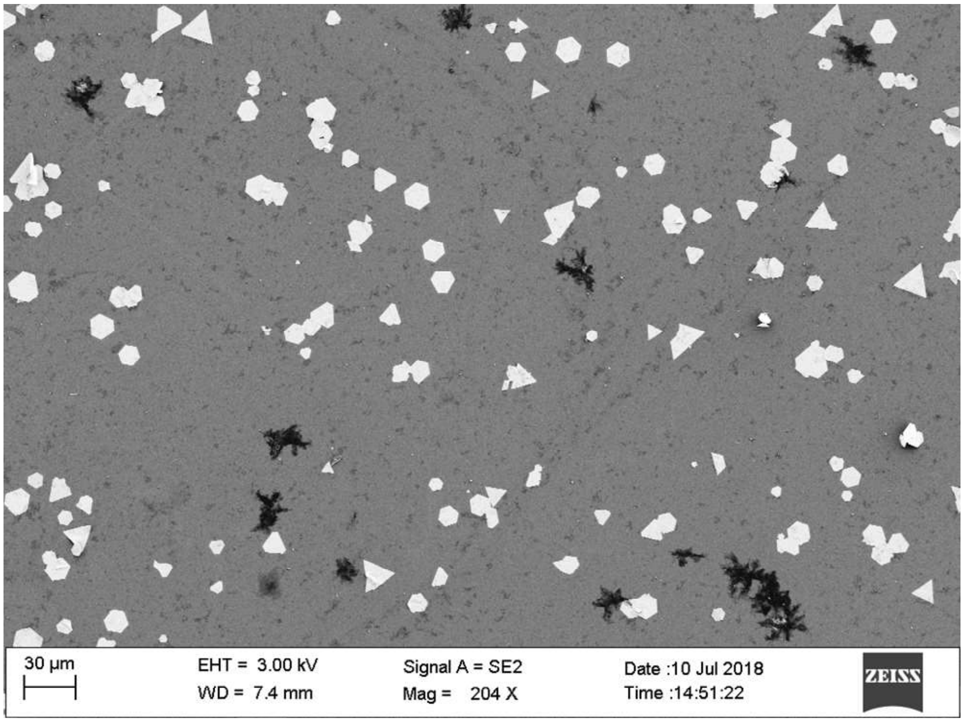

Supplementary Figure 1. SEM characterization. Scanning electron microscopy (SEM) image of the chemically synthesized Au nanoplates where hexagonal and triangular shapes dominated the particles.

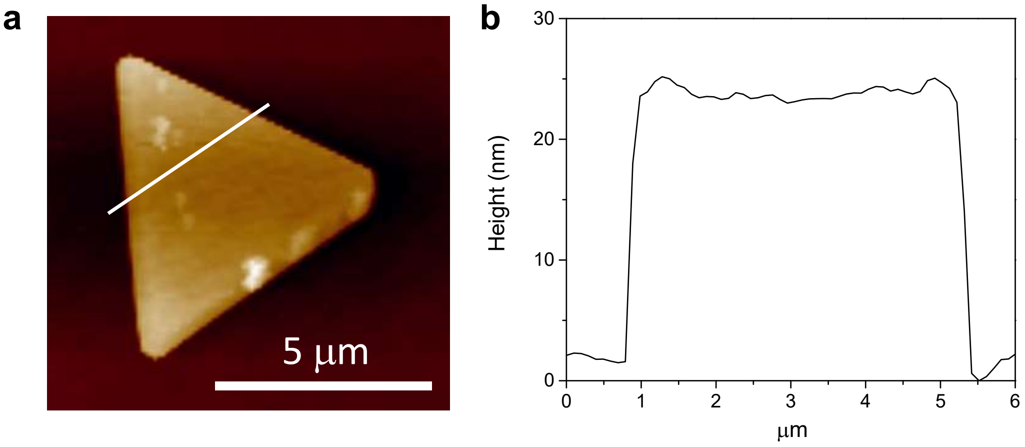

Supplementary Figure 2. AFM characterization. a Atomic force microscopy (AFM) image of a representative Au nanoplate, where the line cut gives the thickness of the nanoplate of ~ 25 nm as shown in **b**.

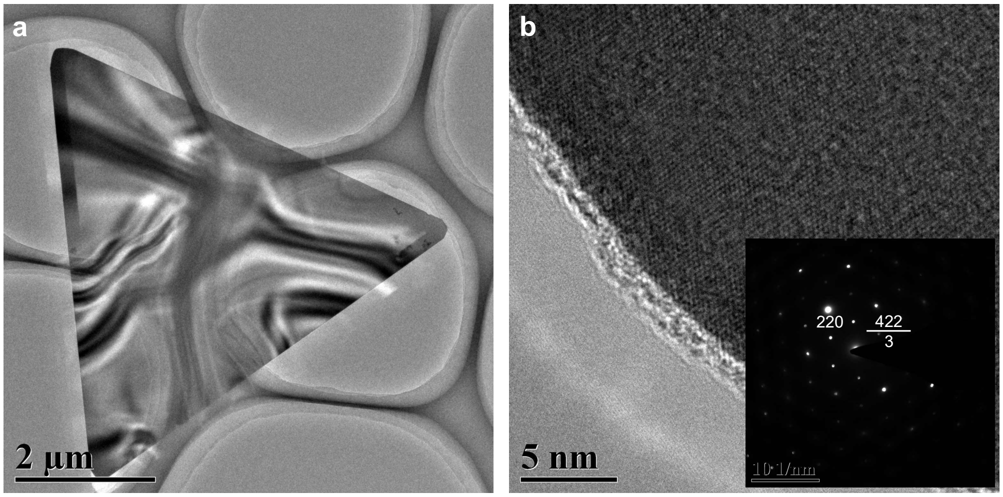

Supplementary Figure 3. TEM characterization. **a** Transmission electron microscopy (TEM) image of a Au nanoplate, and **b** the corresponding high resolution TEM image. The selected area electron diffraction (SAED) pattern indicates the single-crystalline nature of the Au nanoplates with the surfaces being {111} planes. An amorphous surface layer can be seen which is attributed to surfactant capping molecules.

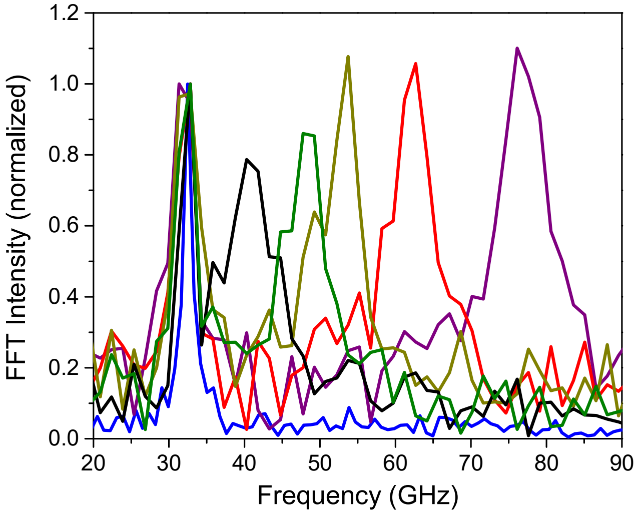

Supplementary Figure 4. FFT spectra of the vibrations of Au nanoplates on glass. Brillouin oscillations and localized acoustic vibrations were observed when placing the Au nanoplates on glass substrates. The localized acoustic vibrations have average quality factor of 10 ± 3 (errors equal the standard deviation).

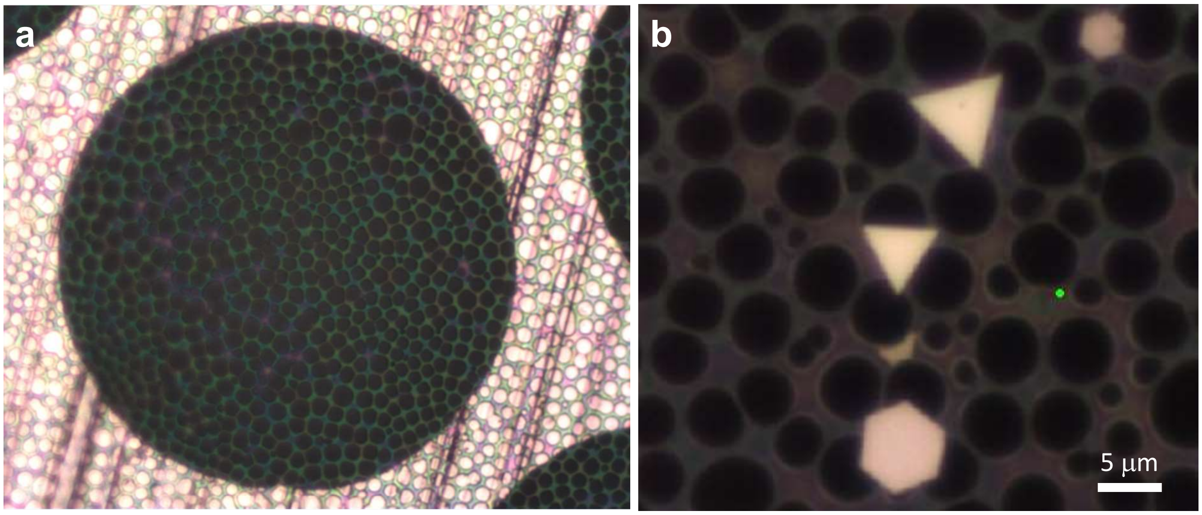

Supplementary Figure 5. Optical images of TEM grid and Au nanoplates. a Optical image of the TEM copper grid where the supported layer was Lacey carbon film. **b** A few Au nanoplates were supported on the film.

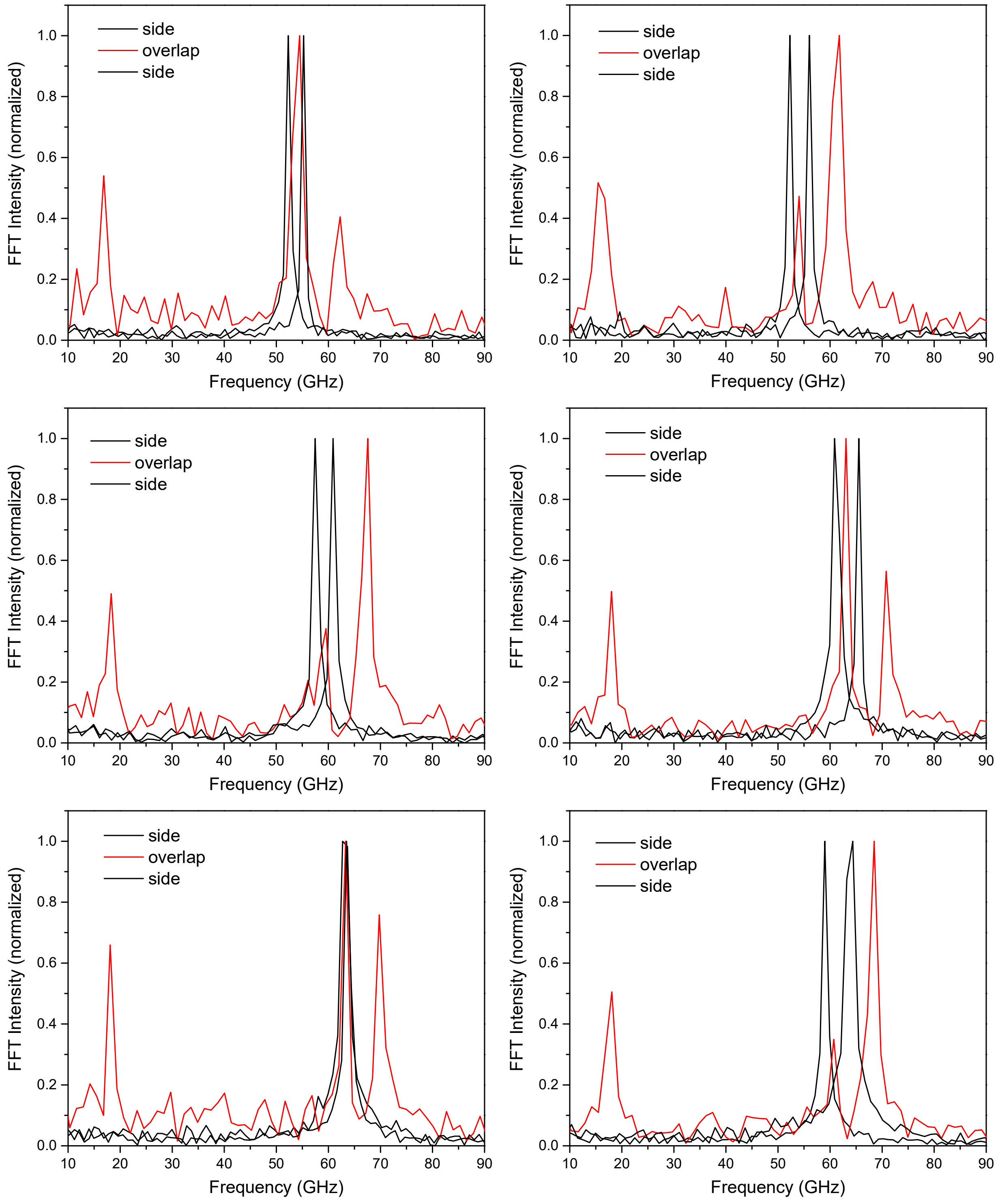

Supplementary Figure 6. Strong vibrational coupling. More vibrational spectra which show strong coupling between Au nanoplates.

Mode f_1 (GHz)	Mode f_2 (GHz)	Coupled f_+ (GHz)	Coupled f_- (GHz)	Coupled f_{rel} (GHz)
29.84	44.24	50.16	33.97	11.28
47.62	59.93	65.89	51.26	18.09
51.35	57.12	64.6	54.26	19.38
51.38	57.38	65.54	55.56	19.38
52.29	55.24	62.26	54.47	16.86
52.29	56.02	61.78	54.05	16.09
52.29	71.26	76.13	55.77	20.1
53.22	55.09	62.02	54.26	16.8
53.66	55.73	62.02	54.85	16.8
55.09	61.8	67.18	58.72	16.8
55.24	69.29	74.28	58.5	18.57
56.18	68.16	74.32	59.36	18.26
57.47	60.92	67.58	59.56	18.33
57.79	62.95	69.5	60.49	18.02
58.05	62.73	70.02	61.44	18.3
58.52	66.48	71.06	62.02	18.09
58.99	63.79	68.48	60.72	18.09
59.01	64.3	69.77	60.72	18.09
60.86	65.54	71.61	63.06	18.23
63.2	63.49	69.77	63.31	18.09

Mode f_1 (GHz)	Mode f_2 (GHz)	Coupled f_+ (GHz)	Coupled f_- (GHz)	Coupled f_{rel} (GHz)
66.48	78.21	84.31	70.04	20.75
70.03	70.96	80.42	70.69	22.05
41.77	61.86	67.58		16.04
42.13	67.42	70.04		16.21
45.48	59.86	62.26		11.45
47.62	56.34	62.66		16.8
51.5	67.23	71.06		15.5
51.82	65.36	69.77		16.8
52.29	62.56	67.18		16.8
53.22	56.02	61.5		16.8
55.09	61.8	65.89		16.8
55.24	71.21	76.52		18.16
57.12	61.8	65.64		14.16
57.89	60.86	67.18		18.09
60.92	65.52	71.02		17.18
67.08	74.87	81.71		20.83
35.48	58.05		37.47	15.5
46.69	61.62		50.18	15.63
49.63	70.22		52.77	18.02
57.12	70.96		59.43	18.09

Supplementary Table 1. Experimental data for coupled Au nanoplates. Fitting errors of the transient absorption trances were estimated to be $< 5 \times 10^{-4}$ (error percentage) due to the high quality factors and large signal to noise ratios.

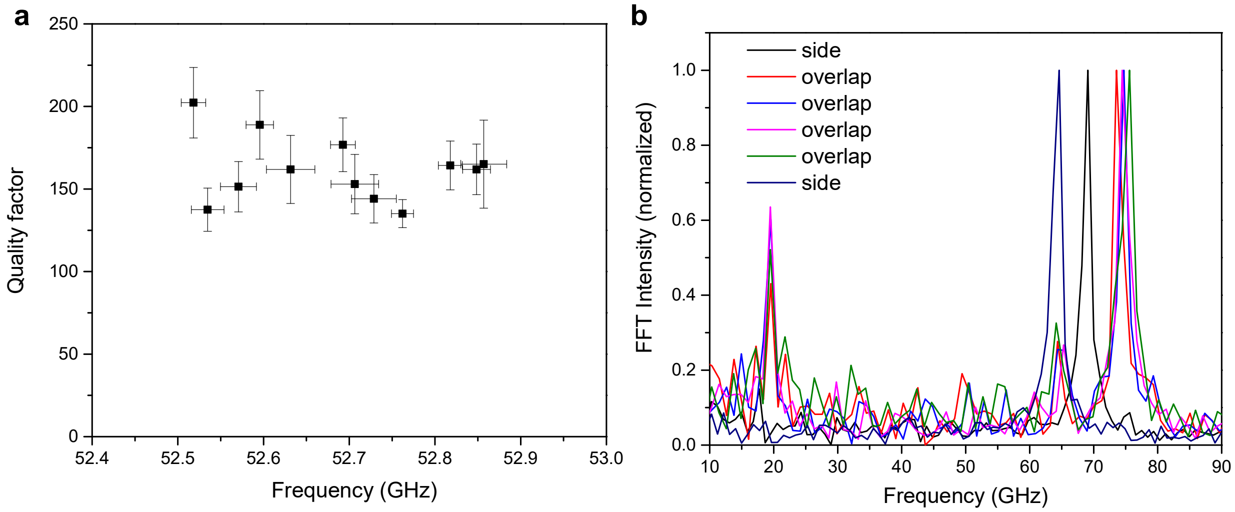

Supplementary Figure 7. System errors determinations. a Statistic analysis of the experimental errors for single vibrating Au nanoplates. The standard deviations of the measured frequencies and quality factors were 52.69 ± 0.12 GHz and 162 ± 20. **b** FFT spectra for two overlapping nanoplates measured in different locations. The frequency and standard deviation for the higher frequency mode of the coupled system are 74.58 ± 0.84 GHz.

Although the relative fitting errors for the individual transient absorption trances were estimated to be $< 5 \times 10^{-4}$ (error percentage), other factors in our experiments affect the reproducibility of the frequencies and lifetimes, such as the setup stability, environment inhomogeneity, PVP molecules between Au nanoplates, etc.. We determined the system errors by measuring the same nanoplate multiple times at different positions. The standard deviation of the measured frequencies for isolated Au nanoplate was 52.69 ± 0.12 GHz (Supplementary Figure 7**a**). Similarly, the standard deviation of the vibrational frequencies for a pair of coupled nanoplates was 74.58 ± 0.84 GHz (Supplementary Figure 7**b**). The large spread of measured coupling frequencies measured for different coupled nanoplates is thus not an instrumental effect, and is probably due to factors such as differences in the amount of PVP between Au nanoplates, that could affect the coupling strength.

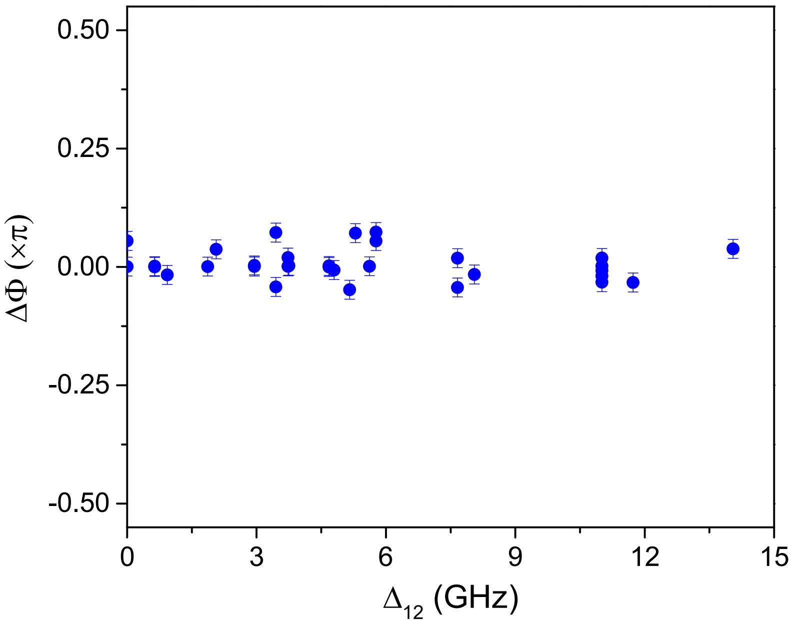

Supplementary Figure 8. Phase difference between the coupled modes. The vibrational phase difference between the coupled modes f_+ and f_-. The error bars were determined from fitting the vibrational traces.

For the coupled Au nanoplates, the phase differences $\Delta\phi$ between the f_+ and f_- were obtained from fitting the time-domain transient absorption traces. The relatively small phase difference indicates that the vibrational modes are normal modes of the system that are excited by ultrafast laser induced heating by the pump laser pulse.

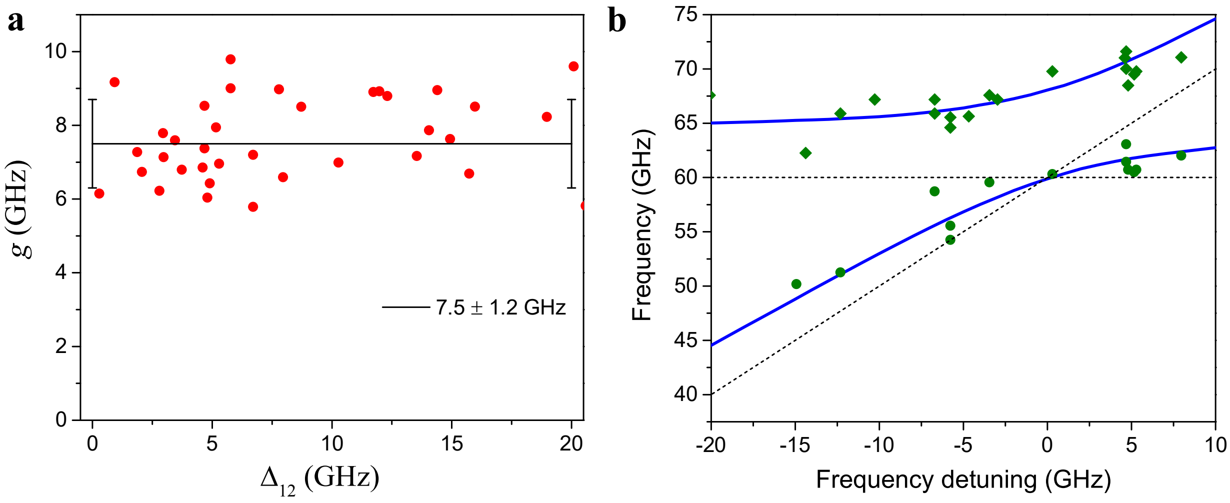

Supplementary Figure 9. Vibrational coupling constant and avoided crossing. a The calculated coupling strength g for the measured data listed in Supplementary Table 1. **b** The mode splitting of the strongly coupled metallic nanoresonators with $f_1 \approx 60$ GHz. The symbols are the experimental data and the solid lines are the theoretical calulcations based on coupled harmonic oscillators with coupling strength $g = 7.5$ GHz.

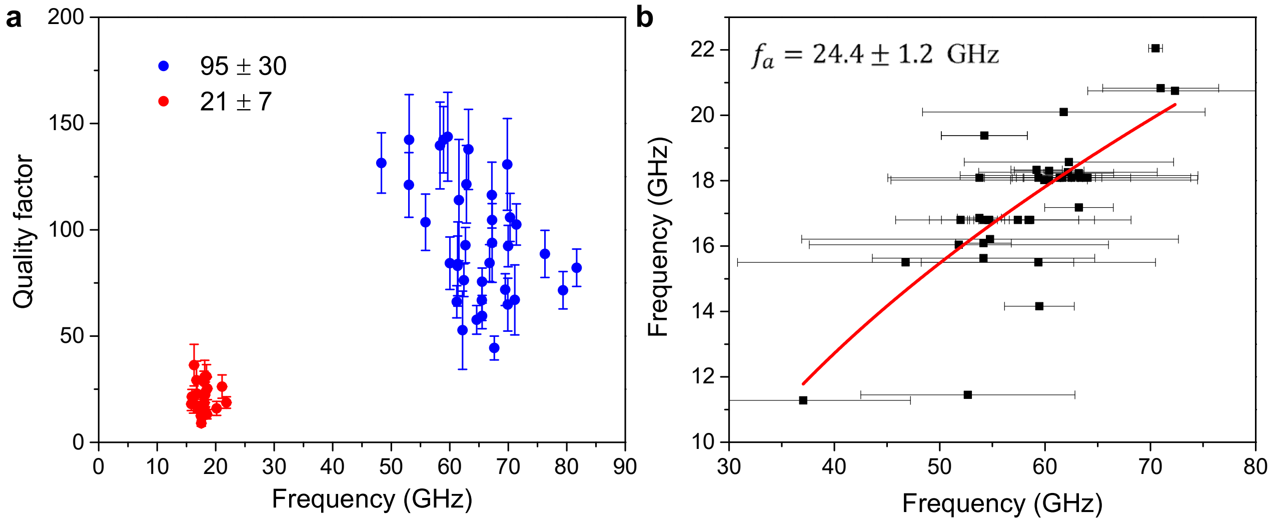

Supplementary Figure 10. Quality factors of the modes in the coupling systems. a Vibrational quality factor of the strongly coupled resonators. The modes f_+ and f_- had values of 95 ± 30 (standard deviations), while the mode that is assigned to the relative motion between the two nanoplates has a smaller quality factor of 21 ± 7 (standard deviations). The error bars in the graph were the fitting uncertainty. **b** Fitting to the relative motion mode to determine the characteristic cut-off frequency f_a, which characterizes the bond spring constant between the Au nanoplates.

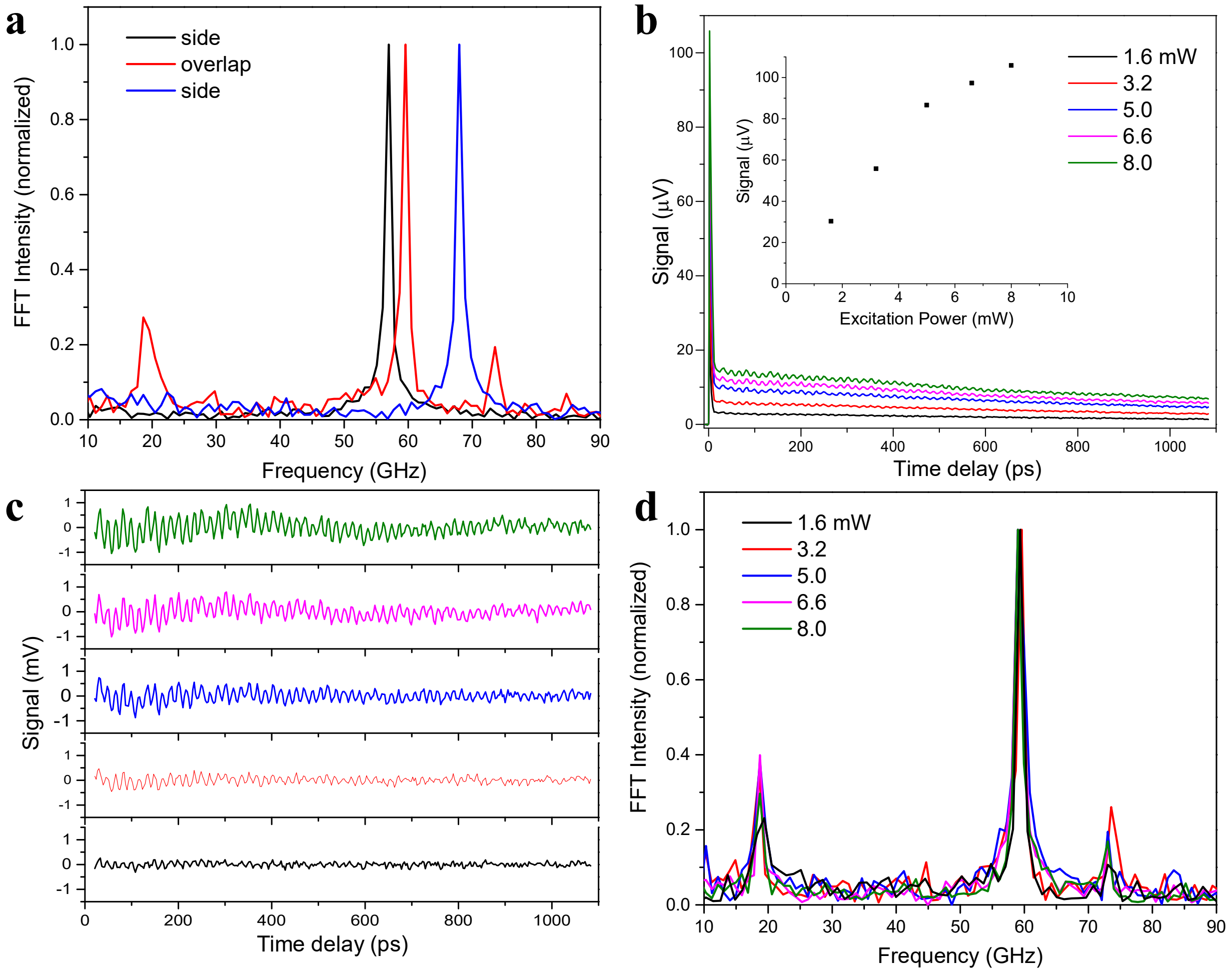

Supplementary Figure 11. Excitation power dependent strong vibrational coupling. a Mechanical coupling between two Au nanoplates. **b** Power dependent transient absorption traces for coupling Au nanoplates probing on the overlapping area. The inset shows the power dependent transient absorption signals. **c** The isolated mechanical vibrations after subtracting the electron-phonon and phonon-phonon contributions to the transient absorption signal. **d** The corresponding FFT of the traces in **c** which indicates the coupling strength was insensitive to the pump power up to 8 mW.

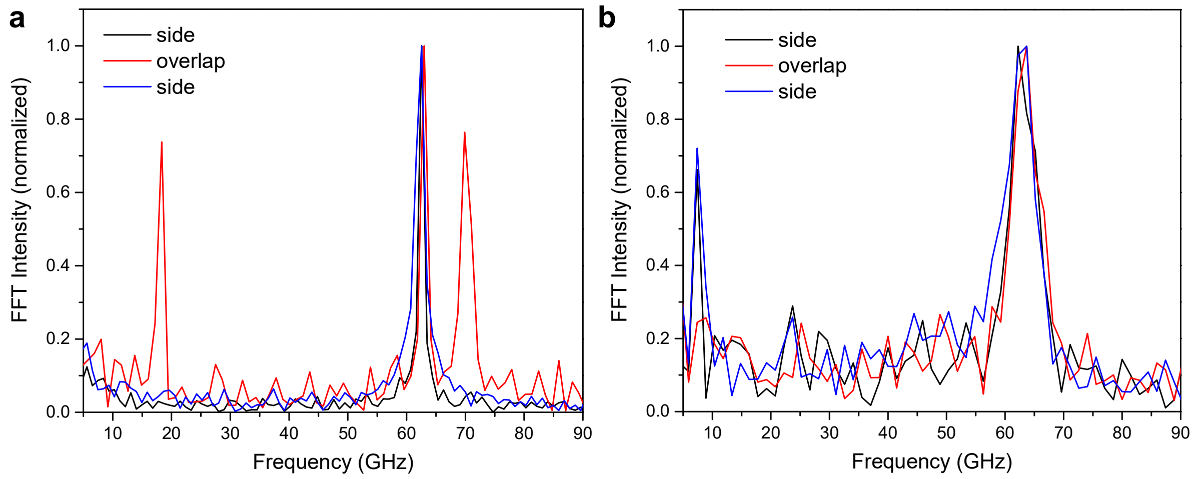

Supplementary Figure 12. Environmental effect on the vibrational coupling. a Spectrum for a strongly coupled resonator. **b** Changing the local environment by adding water completely extinguishes the coupling. The lower frequencies at ~7.4 GHz corresponds to Brillouin oscillations in the water.

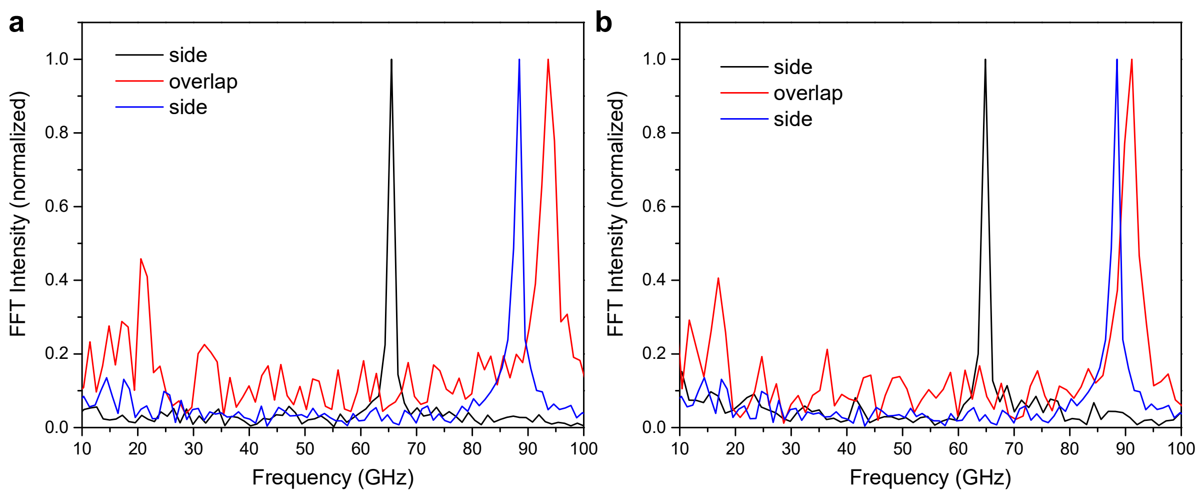

Supplementary Figure 13. Recovery of the vibrational coupling. a Strongly coupled resonators before adding water. **b** Spectrum after adding water to the sample, and then allowing the water to evaporate. The coupling is restored.
